# Analytical performance of a bronchial genomic classifier

**DOI:** 10.1186/s12885-016-2153-0

**Published:** 2016-02-26

**Authors:** Zhanzhi Hu, Duncan Whitney, Jessica R. Anderson, Manqiu Cao, Christine Ho, Yoonha Choi, Jing Huang, Robert Frink, Kate Porta Smith, Robert Monroe, Giulia C. Kennedy, P. Sean Walsh

**Affiliations:** Veracyte, Inc., 6000 Shoreline Ct., Suite 300, South San Francisco, CA 94080 USA

**Keywords:** Percepta, Bronchial genomic classifier, Molecular diagnostic, Lung lesion, Bronchial brushing specimen, Analytical verification

## Abstract

**Background:**

The current standard practice of lung lesion diagnosis often leads to inconclusive results, requiring additional diagnostic follow up procedures that are invasive and often unnecessary due to the high benign rate in such lesions (Chest 143:e78S-e92, 2013). The Percepta bronchial genomic classifier was developed and clinically validated to provide more accurate classification of lung nodules and lesions that are inconclusive by bronchoscopy, using bronchial brushing specimens (N Engl J Med 373:243–51, 2015, BMC Med Genomics 8:18, 2015). The analytical performance of the Percepta test is reported here.

**Methods:**

Analytical performance studies were designed to characterize the stability of RNA in bronchial brushing specimens during collection and shipment; analytical sensitivity defined as input RNA mass; analytical specificity (i.e. potentially interfering substances) as tested on blood and genomic DNA; and assay performance studies including intra-run, inter-run, and inter-laboratory reproducibility.

**Results:**

RNA content within bronchial brushing specimens preserved in RNAprotect is stable for up to 20 days at 4 °C with no changes in RNA yield or integrity. Analytical sensitivity studies demonstrated tolerance to variation in RNA input (157 ng to 243 ng). Analytical specificity studies utilizing cancer positive and cancer negative samples mixed with either blood (up to 10 % input mass) or genomic DNA (up to 10 % input mass) demonstrated no assay interference. The test is reproducible from RNA extraction through to Percepta test result, including variation across operators, runs, reagent lots, and laboratories (standard deviation of 0.26 for scores on > 6 unit scale).

**Conclusions:**

Analytical sensitivity, analytical specificity and robustness of the Percepta test were successfully verified, supporting its suitability for clinical use.

**Electronic supplementary material:**

The online version of this article (doi:10.1186/s12885-016-2153-0) contains supplementary material, which is available to authorized users.

## Background

Lung cancer has the highest mortality of all malignancies with approximately 160,000 deaths per year in the U.S. and a 5-year survival rate of only 17 % [1]. The majority of lung cancers are diagnosed at an advanced stage, although it has been reported that detection at an early stage leads to improved survival. Recommendations call for subjects with a positive radiological imaging finding to be managed according to the likelihood of malignancy [2], with low risk subjects referred for radiological surveillance and intermediate to high risk subjects referred for biopsy procedures. Bronchoscopy is considered the safest biopsy approach and it is estimated that 500,000 bronchoscopies are performed per year in the U.S. [3], of which roughly half are for the diagnosis of lung cancer. However the clinical sensitivity of bronchoscopy is imperfect, particularly with small and peripheral suspicious lesions [4].

It has been shown that exposure to tobacco smoke alters gene expression in airway epithelial cells [5, 6], and that a subset of genes are altered irreversibly [7], establishing a basis for diagnosing smoking related diseases. Subsequent studies showed that gene expression profiling of epithelial cells collected from the main stem bronchus during bronchoscopy can improve the sensitivity of bronchoscopy [8], and more recently using a similar approach, a bronchial genomic classifier (Percepta®) has been validated in large, multicenter, prospective trials [9, 10]. The Percepta test relies on collection of bronchial epithelial cells from a normal appearing area of the mainstem bronchus from subjects undergoing bronchoscopy for suspicion of lung cancer. The test was shown to have a negative predictive value of 91 % with intermediate risk patients and 100 % with low risk patients [9, 10]. The potential utility of the test is therefore to avoid unnecessary invasive diagnostic procedures (such as transthoracic needle biopsy or surgical lung biopsy) and the associated complications when bronchoscopy is inconclusive in patients with benign disease [11, 12].

While the clinical validity of the Percepta test has been demonstrated in independent studies [9, 10], it is equally important to demonstrate analytic validity of this newly-developed molecular test. The Evaluation of Genomic Applications in Practice and Prevention (EGAPP) Working Group and the Centers for Disease Control’s ACCE Project (*A*nalytic validity, *C*linical validity, *C*linical utility and associated *E*thical, legal and social implications) have defined parameters which should be used to evaluate analytical validity of novel genomic tests [13, 14]. Here we report the results of recommended studies designed to test the analytical performance of the Percepta test. Studies included evaluation of specimen stability during collection, shipment and storage, analytical sensitivity to input RNA quantity, analytical specificity in response to contaminating blood and genomic DNA, and several reproducibility studies (intra- and inter-assay, and inter-laboratory), demonstrating robustness to changes across a range of technical variables. Quality control recommendations were extensively implemented and verified via the use of control materials and in-process quality checkpoints at key steps in the Percepta procedure.

## Methods

### Specimens

Normal appearing bronchial epithelial cells (BEC) were collected from the mainstem bronchus using standard cytology brushes during AEGIS 1 and AEGIS 2, two prospective, multicenter, observational studies (NCT01309087 and NCT00746759). The enrolled patients had already been referred for bronchoscopy examinations as part of their clinical care for suspicion of lung cancer. Following sample collection, brushes were immediately clipped and submerged in RNAprotect preservative solution (QIAGEN, Valencia, CA) post-collection. Samples were stored at 2–8 °C before being shipped in a NanoCool shipper (NanoCool, Albuquerque, NM) with active cooling to 2–8 °C, and stored at 2–8 °C upon receipt prior to RNA extraction.

Fresh peripheral blood samples were collected from healthy voluntary participants. Immediately after collection, the blood samples were mixed with the RNAprotect preservative at a 1:5 ratio as recommended by the manufacturer. Subsequently, the pure blood samples were tested following the Percepta molecular test lab procedure as outlined below from total RNA extraction to array results.

Ethics approval was obtained prior to the initiation of the studies described in this report. To characterize analytical performances of the assay, we used total RNA samples (anonymously with no Protected Health Information) that were derived from human bronchial epithelial cell specimens. These total RNA samples were already available from previously registered clinical trials which were published as clinical validation studies [9, 10]. Additionally, we have obtained ethics approval from the Liberty IRB and informed consents from all participants for the use of the freshly collected blood samples.

### RNA extraction, amplification, and microarray hybridization

The Percepta molecular test lab procedure starts with the extraction of total RNA from bronchial brushing specimens using the miRNeasy Kit (QIAGEN, Valencia, CA). Yield was measured using NanoDrop 8000 instruments (NanoDrop, Wilmington, DE) and quality was measured by the RNA Integrity Number (RIN) generated by the BioAnalyzer System (Agilent Technologies, Santa Clara, CA). Samples with concentration <21 ng/μL and/or RIN <4 were stopped from further processing per pre-specified QC criteria. Positive (lung tissue lysate) and negative (water) controls were developed and used as applicable with pre-defined RNA yield and quality acceptance criteria to ensure the reliability of the procedure. For each sample, 200 ng of total RNA were amplified using the Ambion WT Expression Kit (Life Technologies, Carlsbad, CA), fragmented and labeled using the Affymetrix WT Labeling and Controls Kit (Affymetrix, Santa Clara, CA), followed by overnight hybridization of 2.75 μg biotin-labeled cDNA to a Gene 1.0 ST microarray (Affymetrix). The arrays were then washed, stained, and scanned on a GeneChip System GCS3000 or DXv2 (Affymetrix) following manufacturer’s protocols. Cancer positive and cancer negative total RNA controls were included in each sample batch starting from the amplification step. Pre-defined specifications for yield, quality, and Percepta classification of these control samples were used as batch acceptance criteria.

### Genomic DNA analysis

To evaluate the genomic DNA amount present in the total RNA samples, the Quantifast SYBR Green PCR kit was used following manufacturer’s protocol (QIAGEN, Valencia, CA), using a normalized 50 ng total RNA input for the test samples.

### Data analysis

All data analysis was done in R version 3.1.2 [15]. To obtain a Percepta test result, transcript signal intensities for each array were first normalized using frozen robust multi-array analysis (fRMA) [16]. The Percepta calls (two-class calls based on a locked score decision boundary) and scores were subsequently derived using 23 genes and the patient age following the classifier algorithm as described [10]. Note that in the previous study [10], the Percepta classifier score refers to a prediction from logistic regression which returns predicted probabilities of lung cancer within a range of 0 to 1. In this report, a logit transform was applied to the scores so that their range would be appropriate for linear model fits in the statistical analyses that follow. This is explained in greater detail in Additional file 1.

Brushing sample stability was established using an ANOVA test of means of yield and failure rates of RIN over time. Linear mixed effect models were used to evaluate the effects of RNA input amount, blood interference, and genomic DNA interference at the 5 % significance level. To claim that a variable was not statistically significant, its *p*-value had to be >0.05 under all attempted models, and the lowest *p*-value was reported so that lack of significance was ensured for all models fit. Transcript signal intensity Pearson correlations between arrays from different test sample groups were calculated from the genes used in the algorithm. All 95 % confidence intervals for standard deviations (SD) were obtained by bootstrap where the residuals of a linear mixed-effects model controlling for sample and other sources of variation (depending on the type of SD reported) were sampled with replacement to create a bootstrap sample. The distribution of blood contamination level in the total RNA from the AEGIS clinical samples was simulated based on 1) the observed blood contamination levels based on a predefined color scale, 2) the mass variation of pure fresh blood derived total RNA, and 3) the mass variation of total RNA from the clinical samples, assuming these three factors contribute to the blood contamination level independently. The simulation to assess the maximum tolerable level of variation in Percepta scores was performed by making multiple random draws from a normal distribution for each AEGIS sample, with the mean defined as the Percepta score obtained during the clinical validation [9, 10] and the SD at each specified level. The resulting average performance from all draws (each draw across all samples) at a given SD level were evaluated for sensitivity, specificity, NPV and PPV. Further details of the data analysis can be found in Additional file 1.

## Results

### Control materials

Multiple lots of lung tissue lysate were manufactured (at Veracyte) and used as process controls during RNA extraction. Three different lots of controls were tested over several weeks of independent runs with 7 replicates of each lot per run, by two different operators. Testing of three lots is standard practice to verify the reproducibility of a manufacturing or laboratory process. Lung lysate controls consistently produced the expected quantity and quality of total RNA, resulting in within-lot coefficients of variation (CV) ranging from 4 to 6 % for yield and 2–4 % for RIN (data not shown).

Similarly, multiple lots of cancer positive and cancer negative total RNA were manufactured (at Veracyte) and tested for their use as process controls for amplification and hybridization steps. The reproducible Percepta results obtained from these controls enabled concurrent monitoring of assay performance for each run. All Percepta tests and studies outlined below included at least one cancer positive and one cancer negative total RNA controls.

### Bronchial brushing specimen stability

**Fig. 1:**
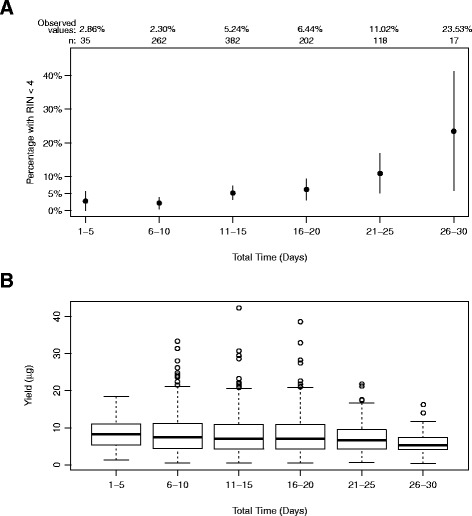
Bronchial brushing specimen stability at 2–8 °C in RNAprotect. All samples from the AEGIS 1 and 2 clinical studies were analyzed at the time of RNA extraction. The resulting QC data were plotted as a function of the cumulative total storage time at 2–8 °C. The number of samples in each time window (n) for both RIN and yield is shown at the top. **a** RIN failure rate. Black dots: observed values. Vertical lines: 95 % CI. **b** Boxplots of total RNA yield

To demonstrate the cumulative stability of the RNA content within the preserved BEC samples under the typical collection and storage conditions, comprehensive sample tracking data were collected from AEGIS samples and the stability was evaluated using metrics of RNA yield and integrity. The length of time from sample collection till RNA extraction was accounted for. With the pre-specified sample quality criteria, no statistically significant difference was observed among samples with a cumulative 2–8 °C storage time of up to 20 days based on RIN failure rate (*p* = 0.148, Fig. 1a) or RNA yield (*p* = 0.955, Fig. 1b). Combined with the manufacturer’s recommended 2–8 °C storage of up to 4 weeks, these data strongly support the sample storage stability in RNAprotect at 2–8 °C at the clinical site and testing lab and shipping in chilled box for routine practice.

### Analytical sensitivity - total RNA input quantity

**Fig. 2:**
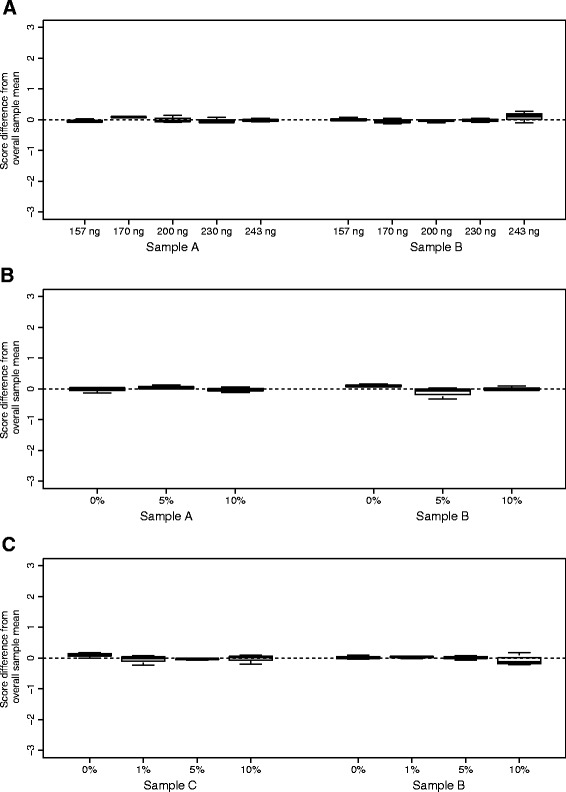
Analytical sensitivity and specificity of the Percepta test. The y-axes are on a relative scale, with 0 representing the mean of each sample across all input levels (mean centered). Sample A and C are cancer negative. Sample B is cancer positive. Each box represents test results from technical triplicates. **a** Effect of input mass variation on Percepta score. **b** Analytical specificity of the Percepta test against blood. The x-axis shows the percentage of total input mass, fixed at 200 ng, from the blood. **c** Analytical specificity of the Percepta test against genomic DNA. The x-axis shows the percentage of total input mass, fixed at 200 ng, from genomic DNA

While the standard total RNA input quantity to the Percepta assay is fixed (200 ng), concentration measurement (by NanoDrop) and pipetting (using Rainin LTS pipets) variability around this nominal input amount are expected in routine practice. Therefore, a study was performed to characterize the transcript array signal intensities and Percepta results relative to variability in total RNA input quantity. Based on the manufacturers’ specifications in concentration measurement and pipetting, the standard deviation (SD) of such variation translates to 15.2 ng, given the intended 200 ng input. Therefore, the titration levels were designed to be 200 ng ± 30 ng (corresponding to ± 1.96 SD, covering 95 % of the samples) and 200 ng ± 43 ng (corresponding to ± 2.81 SD, covering 99.5 % of the samples). Total RNA from a cancer positive and a cancer negative bronchial brushing sample were processed in triplicate through the Percepta test at the designed total RNA input levels (157, 170, 200, 230 and 243 ng). As shown in Fig. 2a, Percepta scores for each sample did not differ significantly with RNA input when evaluated with a linear mixed effect model (*p*-value = 0.69). The transcript signal intensities of each sample were equally highly correlated within each single group of RNA input (Pearson R^2^ coefficients 0.986–0.998, with a mean of 0.992) and between the test input groups and the baseline 200 ng condition (R^2^ coefficients 0.982–0.998, with a mean of 0.992). Overall, this study demonstrated highly robust analytical sensitivity of the Percepta test to RNA input quantity variation within the tested range.

### Analytical specificity - blood

Occasionally, bronchial brushing samples may contain small amounts of blood due to variation in the collection procedure or to individual patients. This was confirmed by the visual inspection of samples collected during the AEGIS trials, which showed that greater than 80 % of the samples have no visible blood contamination. The procedure to collect BEC specimens limits the volume of contaminating blood using standard sheathed cytology brushes (Additional file 1). Further, a simulation of the distribution of blood contamination levels in the total RNA from the clinical samples showed that <1 % of the clinical samples have >1 % of blood derived RNA, with the most extreme cases have ~10 % of blood derived RNA (Additional file 1). To experimentally test the impact of blood on the Percepta results, in vitro mixtures were created using RNA from one cancer positive or one cancer negative brushing sample that were each spiked with the total RNA derived from a fresh whole blood sample, while maintaining the combined total RNA input mass at 200 ng. Figure 2b shows that when 5 and 10 % of blood derived total RNA were spiked into the brushing derived RNA samples and tested in triplicates via the Percepta test, no score shifts were observed compared to 0 % blood (*p*-value = 0.515), supporting that the Percepta test is robust against blood contamination.

### Analytical specificity – genomic DNA

Genomic DNA was tested as a potentially interfering substance, as presence of DNA can occur from inadvertent variation or deviations from the RNA extraction process. To assess the extent of genomic DNA contamination, a qPCR based genomic DNA quantitation assay was used. A set of planned RNA extraction procedural deviation tests revealed that excessive genomic DNA carryover was only observed when chloroform was omitted in the procedure, which was a visually detectable deviation. For all other deviation conditions tested, the observed genomic DNA levels in the RNA samples were consistently 1 % or less. Similarly, the levels of contaminating DNA in samples isolated during the AEGIS trials were also found to be consistently <1 % (data not shown). Thus, assay testing was designed for up to 10 % genomic DNA contamination as a worst-case scenario (10 times above baseline). One cancer positive and one cancer negative brushing sample that were tested to have < 1 % genomic DNA were spiked with 1, 5 and 10 % additional genomic DNA and tested via Percepta in triplicate per condition. There was no significant difference in the Percepta score between samples with up to 10 % genomic DNA and samples with no additional genomic DNA spiked in (*p*-value = 0.20) (Fig. 2c). This study demonstrated that the Percepta test is not affected by genomic DNA at the levels encountered in clinical samples.

### Assay reproducibility

In order to assess the maximum tolerable level of variation in Percepta scores, a simulation study was performed by adding increasing levels of random variation *in silico* to the original Percepta scores obtained from the validation samples [9]. The resulting Negative Predictive Value (NPV) was evaluated since the Percepta test is designed to be a high sensitivity (rule-out) test. The simulation demonstrated that the Percepta scores can tolerate a standard deviation of up to 0.4 units on a roughly 6-point scale in order to maintain an NPV of 90 % (Additional file 1).

**Fig. 3:**
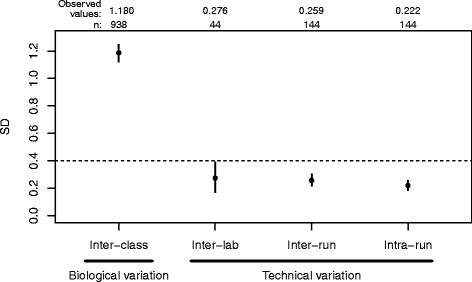
Comparison of Percepta score variability. The inter-class score SD includes biological variation between cancer and non-cancer samples and was computed from all samples passing quality control criteria from the AEGIS 1 and 2 clinical studies. Dashed line: the maximum tolerable level of variation in Percepta scores derived from simulation. Black dots: observed values. Vertical lines: 95 % CI. The number of data points used to calculate each SD (n) is shown at the top

The within-run and inter-run reproducibility of the Percepta test were evaluated using total RNA from 10 bronchial brushing samples with high, medium and low scores, and 6 control samples, processed in triplicate in three experimental runs (144 Percepta results), varying reagent lots, operators, and days (spanning three weeks). The pooled within-run SD of Percepta scores was estimated to be 0.222 (95 % CI 0.186 to 0.257; Fig. 3). The transcript signal intensities from within-run replicates had mean R^2^ coefficients of 0.985 (range 0.945 to 0.998).

Additionally, the Percepta scores were estimated to have an inter-run pooled SD of 0.259 (95 % CI 0.217 to 0.304; Fig. 3) across all samples in this study. When replicates were pooled across runs, all brushing samples and total RNA control samples had standard deviations below the aforementioned tolerance of 0.4 units (range 0.082 to 0.340). The transcript signal intensities from across-run replicates had mean R^2^ coefficients of 0.983 (range 0.933 to 0.998). Thus, the Percepta test demonstrated sufficiently high reproducibility across reagent lots, operators, and processing runs. In contrast, the inter-class score SD was estimated to be 1.180 (95 % CI 1.115–1.246), which includes biological variation between cancer and non-cancer samples from the AEGIS 1 and 2 clinical studies.

### Inter-laboratory reproducibility

Total RNA from 22 different patient bronchial brushing samples was processed using the Percepta test in the laboratory where the test was developed (Veracyte Research & Development Laboratory). A second aliquot of RNA from the same samples was later tested in a different, CLIA-certified reference laboratory using different operators, reagent lots, and equipment (same model equipment, different by serial number; Veracyte CLIA laboratory). The Percepta calls for all samples were 100 % concordant between the two laboratories. Further, the Percepta scores of the 22 samples between the two laboratories are highly correlated (R^2^ = 0.992), demonstrating inter-laboratory reproducibility and accuracy of Percepta results. Inter-laboratory pooled standard deviation of Percepta scores was estimated to be 0.276 (95 % CI 0.172–0.389), which is in agreement with the SD of 0.259 calculated for within-lab inter-assay reproducibility. Similarly, transcript signal intensities were highly correlated between laboratories across all samples (median R^2^ 0.984, range 0.960–0.992), consistent with expectations for inter-assay results.

## Discussion

Analytical and clinical validity are important factors in the evaluation of any new molecular test. The clinical validity of the Percepta classifier was previously reported as a useful tool in the clinical evaluation of lung lesions suspected to be cancer [9, 10]. Here we set out to verify the analytical validity of this test. In addition to salient wet-lab studies, *in silico* simulations and modeling were also used as applicable to establish the validity of test criteria.

The entire process from sample collection, storage, shipping, sample processing and classification was evaluated. It was demonstrated that nucleic acids extracted from clinical brushing specimens are stable and yield reproducible results across a variety of conditions. The assay was also shown to be robust to routine RNA input quantity variations.

Analytical specificity was evaluated. From data collected in the prospective clinical studies, it was shown that routine clinical samples contain little to no blood content. Further, in controlled experiments, up to 10 % blood derived RNA showed no impact to the Percepta calls and scores. The Percepta test also showed robustness to potential contaminating genomic DNA. The RNA extraction method used in the test demonstrated consistently low genomic DNA content (<1 %), and up to 10 % genomic DNA spiked into the starting RNA sample have no detectable impact to the Percepta scores.

Analytical reproducibility was evaluated following technical assessment criteria outlined by EGAPP and ACCE, using clinical samples with Percepta scores covering the entire range and concentrated around the decision boundary of the assay [17]. It has been argued that accuracy studies for multi-gene molecular tests are often impossible due to the absence of reference methods [18]. To establish accuracy of the test offered at the CLIA-certified laboratory, it was demonstrated with an inter-laboratory reproducibility study that the results in this lab are identical to those generated in the laboratory where the test was developed. When taken together with the clinical validation studies, the Percepta test successfully achieves EGAPP level I analytic validity criteria. Namely, technical validation involved the extensive use of well-characterized samples with multiple reference standard comparison methods including cytopathology, histopathology, and reference laboratory.

## Conclusion

The robustness of the Percepta test to induced variables, including those that may be encountered in clinical samples, supports that routine testing of bronchial brushing specimens can be achieved at high confidence from the standpoint of analytical performance and reproducibility.

## Additional file

Additional file 1: Supplementary Information. (DOCX 26 kb)

## Abbreviations

ACCE: Analytic validity, Clinical validity, Clinical utility and associated Ethical, legal and social implications
CI: Confidence interval
CLIA: Clinical laboratory improvement amendments
CV: Coefficients of variation
NPV: Negative predictive value
RIN: RNA integrity number
RNA: Ribonucleic acid
SD: Standard deviation
