# Supplementary material for: Analytical performance of a bronchial genomic classifier
Source: BMC Cancer. 2016 Feb 26;16:161. doi: 10.1186/s12885-016-2153-0 (PMC4768333; doi:10.1186/s12885-016-2153-0)
Supplement: Additional file 1: — Supplementary Information. (DOCX 26 kb) [file 12885_2016_2153_MOESM1_ESM.docx]

**Additional file 1**

**Percepta classifier scores: logit transformation**

The Percepta classifier is based on a logistic regression model:

$logit(P\left( Y_{i}=1 | x_{1i}, x_{2i}, \ldots,x_{ki} \right))= \beta_{0}{+\beta}_{1}x_{1,i}{+\ldots\beta}_{k}x_{k,i}$,

where $Y_{i}$ is dichotomous and equals 1 if person *i* has lung cancer, and 0 otherwise; $x_{j,i}$ is the value of predictor *j* for person *i*; and the logit function is defined as $logit\left( x \right)=\ln\left( \frac{x}{1-x} \right)$ for *x* in [0, 1]. This function is one-to-one and monotonic in *x.* In the previous study [3], the Percepta classifier score refers to the predicted values of $P\left( Y_{i}=1 | x_{1i}, x_{2i}, \ldots,x_{ki} \right)$, which fall in the interval [0, 1]. In this publication, the Percepta score refers to $logit(P\left( Y_{i}=1 | x_{1i}, x_{2i}, \ldots,x_{ki} \right))$. This is done to obtain unbounded, continuous values that can be used as dependent variables in further linear model fits.

**Bronchial brushing stability:**

To evaluate the stability of bronchial brushing samples with respect to storage time, the AEGIS data were binned into six groups of five days, as shown in the table below. Seven samples had a total storage time past 30 days, and were removed from the analysis as outliers.

| **Storage time (days)** | **Percentage with RIN > 4** | **Number of samples** |
| --- | --- | --- |
| 1 – 5 | 97.14% | 35 |
| 6 – 10 | 97.70% | 262 |
| 11 – 15 | 94.76% | 382 |
| 16 – 20 | 93.56% | 202 |
| 21 – 25 | 88.98% | 118 |
| 26 – 30 | 76.47% | 17 |

An ANOVA analysis was performed on an indicator variable for RIN > 4 vs. the six bins of total storage time. The same ANOVA analysis was then done on three subsets of the data--the data set with binned total storage time up to 20 days, 15 days, and 10 days--for a total of four model fits. As the results below show, up to 20 days, the percentage of samples with RIN > 4 is statistically indistinguishable across storage times.

| **Maximum storage time** | **p-value** |
| --- | --- |
| 30 days | 0.0003 |
| 20 days | 0.1480 |
| 15 days | 0.1640 |
| 10 days | 0.8390 |

**Blood content simulation**

A standard sheathed cytology brush end is a 10 mm long brush with a metal wired core of ~0.4 mm in diameter (Hobbs Medical Inc. Cat. No. 4206). After collection, the brush is retracted into the plastic sheath which has an inner diameter of ~1.0 mm. Therefore, the theoretical maximum amount of liquid that can be trapped in a single brush without including the plastic brush hairs is calculated as

$$V=\pi{(\frac{1.0}{2})}^{2}\times10-\pi\left( \frac{0.4}{2} \right)^{2}\times10=6.6 \mu L$$

The standard Percepta sample collection procedure requires two brushes to be collected, yielding a theoretical maximum amount of blood carryover of 13.2 μL.

AEGIS samples were given a score of 0 to 3 for the degree of blood contamination in the sample detectable by visual inspection, with 0 indicating no blood and 3 indicating heavy contamination. For simplicity, the volume of blood required to produce different levels of contamination was also estimated, with blood score of 3 corresponding to the theoretical maximum volume calculated as above. As the table below shows, the vast majority of samples had no visible blood contamination.

| **Blood score** | **Observed percentage** | **Estimated blood volume (µL)** |
| --- | --- | --- |
| 0 | 80% | 0 |
| 1 | 15% | 1 |
| 2 | 4% | 4 |
| 3 | 1% | 13.2 |

The percentage of blood contamination by mass is simulated by calculating the blood RNA mass in the total RNA mass (from the clinical specimen). The blood RNA mass was simulated by estimating a blood contamination volume according to the frequencies in the table above, and a blood RNA mass from those observed in a blood collection study. Total RNA mass was based on clinical specimens observed in the AEGIS studies.

A total of 1,000,000 simulated blood contamination percentages were generated in this way. Over 99% had < 1% blood contamination. Less than 0.01% of the simulated values indicated blood contamination of >5%.

**Assessing the effects of RNA input amount, blood, and genomic DNA contamination**

Three separate experiments were done to examine the robustness of the Percepta score to total RNA input amount, blood contamination, and genomic DNA contamination. For each patient and level of the variable of interest, three replicates were run to array.

Two linear mixed effect models were fit to assess the effect of total RNA input amount on the Percepta score. Both models include a random intercept for patient and a term to model the RNA input amount. In the first model, RNA input amount is treated as a categorical variable; in the second, as a continuous numeric variable.

The same analyses were done for the studies assessing the effects of blood contamination and genomic DNA on the Percepta score. Again, two models were fit, both containing a random intercept for patient: the first treated blood contamination or genomic DNA as a categorical variable; the second as a continuous variable.

The table below provides p-values from both model fits for each of the three studies. The patient-specific effect was removed to focus the analysis on non-sample related effects. For the continuous model, the p-value reported is from the regression coefficient t-test. For the categorical model, the p-value reported is from the ANOVA F-test. In the manuscript, the lesser of the two is reported. No significant effect on the Percepta score was observed by the tested levels of input amount, blood contamination, or genomic DNA contamination.

|  |  | **p-values from model fits:** | |
| --- | --- | --- | --- |
|  |  | Model 1 (categorical) | Model 2 (continuous) |
| **Study** | Input amount | 0.692 | 0.738 |
|  | Percentage blood | 0.575 | 0.515 |
|  | Percentage gDNA | 0.500 | 0.200 |

**Assay and inter-laboratory reproducibility**

*Determining tolerance of score variation:*

To determine the maximum tolerated variation measured as score SD, new sets of scores with pre-determined SDs were simulated from a normal distribution and modeled after the AEGIS data. Specifically, the score for each sample in the AEGIS data set was denoted by $z_{i}$, i = 1,…,n. Then, for each sample *i* and standard deviation $\sigma$ in 0.00, 0.02, 0.04,…, 0.60, 100 instances were generated as

$\hat{z}_{i,1},\ldots,\hat{z}_{i,100}\sim N(z_{i}, \sigma^{2})$.

For each standard deviation, the 100 sets of scores were pooled to compute the negative predictive value (NPV). The NPV was calculated using prevalence from the AEGIS intermediate risk cohort [1].

*Estimating intra-run, inter-run, and inter-laboratory variability*:

The pooled intra-run and inter-run standard deviations were computed from the same data set with 10 bronchial brushing samples and 6 controls, processed in triplicate over three experimental runs. The pooled intra-run SD is the residual standard error after modeling the Percepta score on sample, experimental run, and an interaction term. The pooled inter-run SD is the residual standard error after modeling the Percepta score on sample.

The inter-laboratory pooled SD was computed from a separate study with 22 bronchial brushing samples. The inter-laboratory pooled SD is the residual standard error after modeling Percepta score on sample.

Confidence intervals for each of these standard deviations were obtained via the semi-parametric bootstrap. The procedure can be summarized as follows: the residuals from each model fit are sampled with replacement, and the residual standard error was computed directly, using the fact that it is the root mean square of these deviations (where the denominator in the mean is the appropriate degrees of freedom). This was repeated 1000 times to create a bootstrap sample of 1000 standard deviations. Letting $q_{2.5\%}$ and $q_{97.5\%}$ denote the 2.5^th^ and 97.5^th^ quantiles of the bootstrap sample and $\hat{\theta}$ our original SD estimate, and 95% confidence interval was defined as$(2\hat{\theta}-q_{97.5\%}, 2\hat{\theta}-q_{2.5\%})$, a.k.a. the basic bootstrap [2].

*Transcript signal intensity correlations*:

Reproducibility was also assessed by computing the correlation between two assays, restricted to the 23 genes in the Percepta model. Correlation using all >32,000 genes measured by the assay was also computed, and found to be similar to the correlations computed with only 23 genes: all were very high and close to 1. When they did differ, correlation using genes from the entire assay was usually slightly higher. Correlations using 23 genes were reported in the manuscript because assessing similarity in the expression levels of these particular genes was more relevant to the performance of the Percepta model.

**References**

1. Silvestri, G. A., Vachani, A., Whitney, D., Elashoff, M., Porta Smith, K., Ferguson, J. S., Spira, A.. A Bronchial Genomic Classifier for the Diagnostic Evaluation of Lung Cancer. New England Journal of Medicine. 2015; 373(3):243-251
2. Davison, A. C., and D. V. Hinkley. "Bootstrap Methods and Their Applications." (1999).
3. Whitney DH, Elashoff MR, Porta-Smith K, Gower AC, Vachani A, Ferguson JS, et al. Derivation of a bronchial genomic classifier for lung cancer in a prospective study of patients undergoing diagnostic bronchoscopy. BMC Med Genomics. 2015; 8: 18
